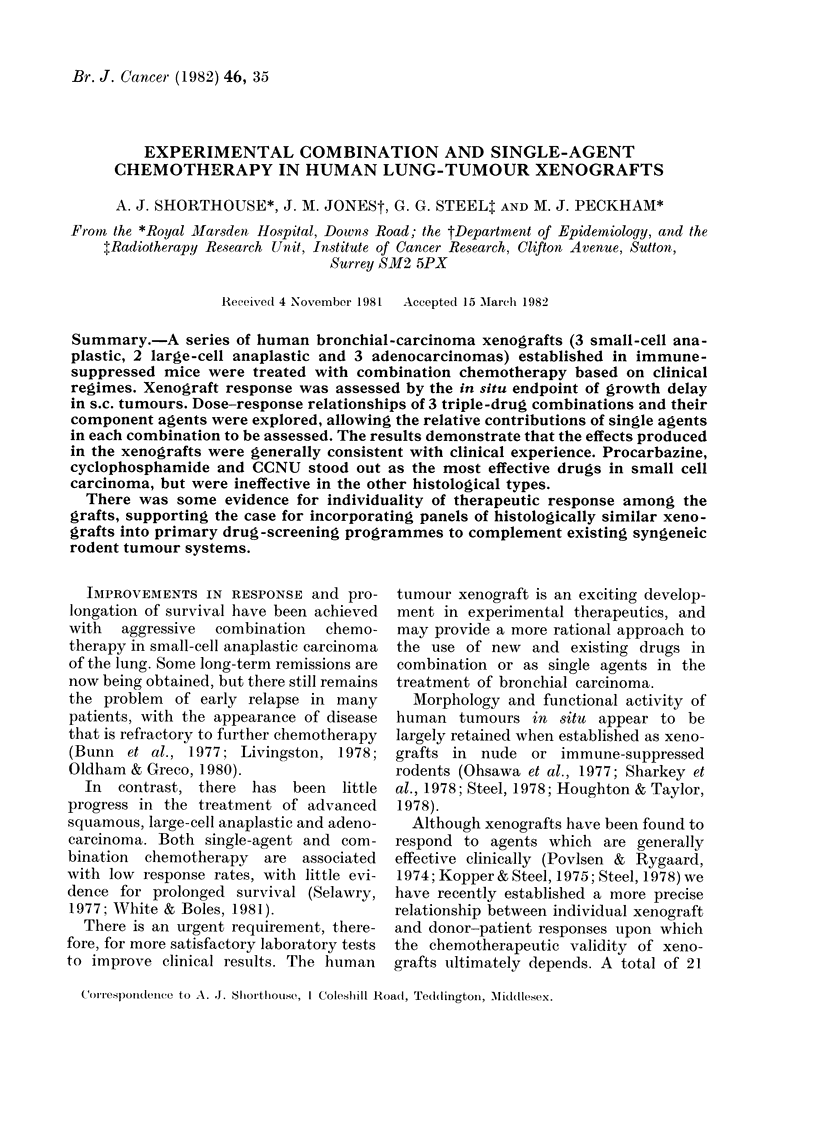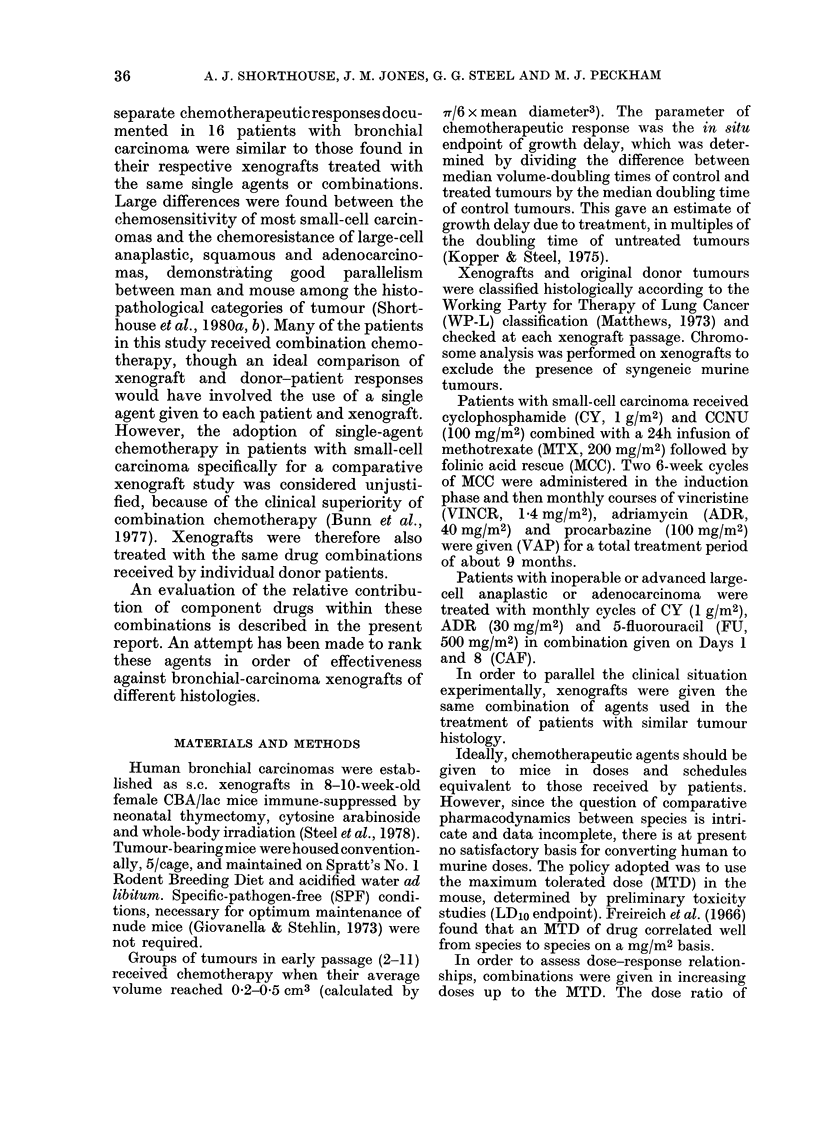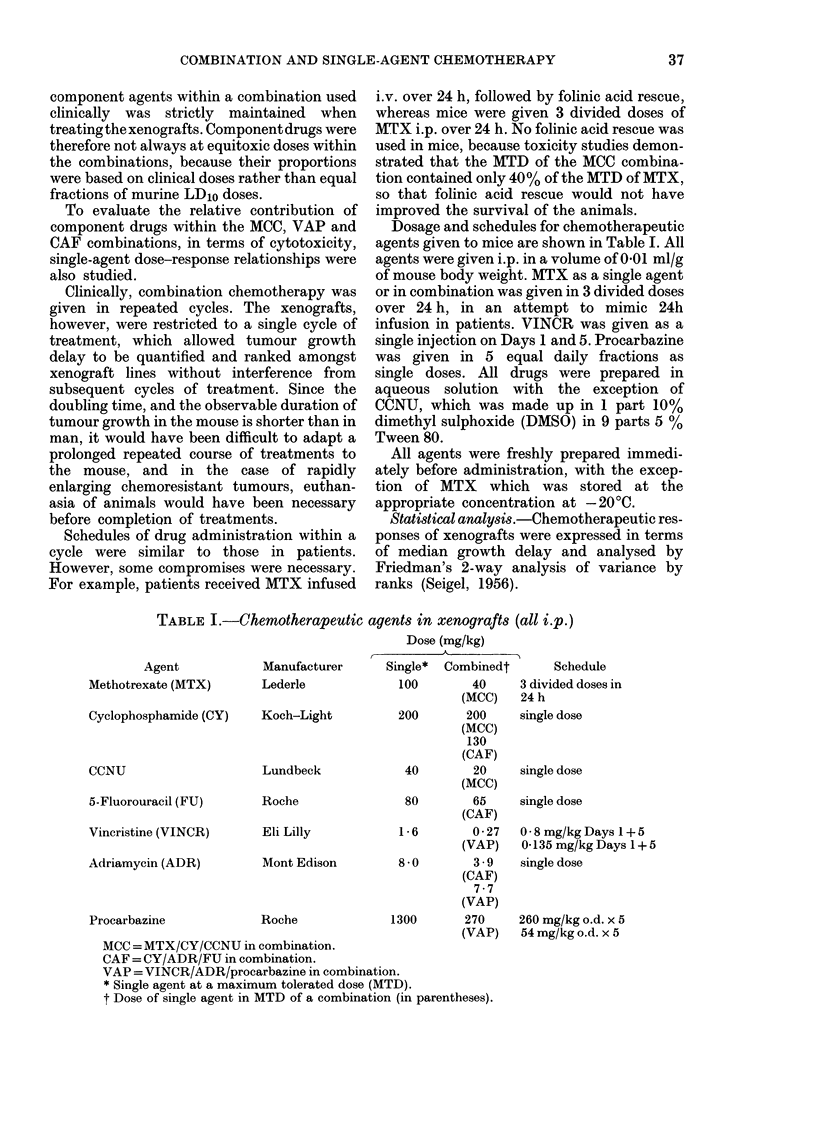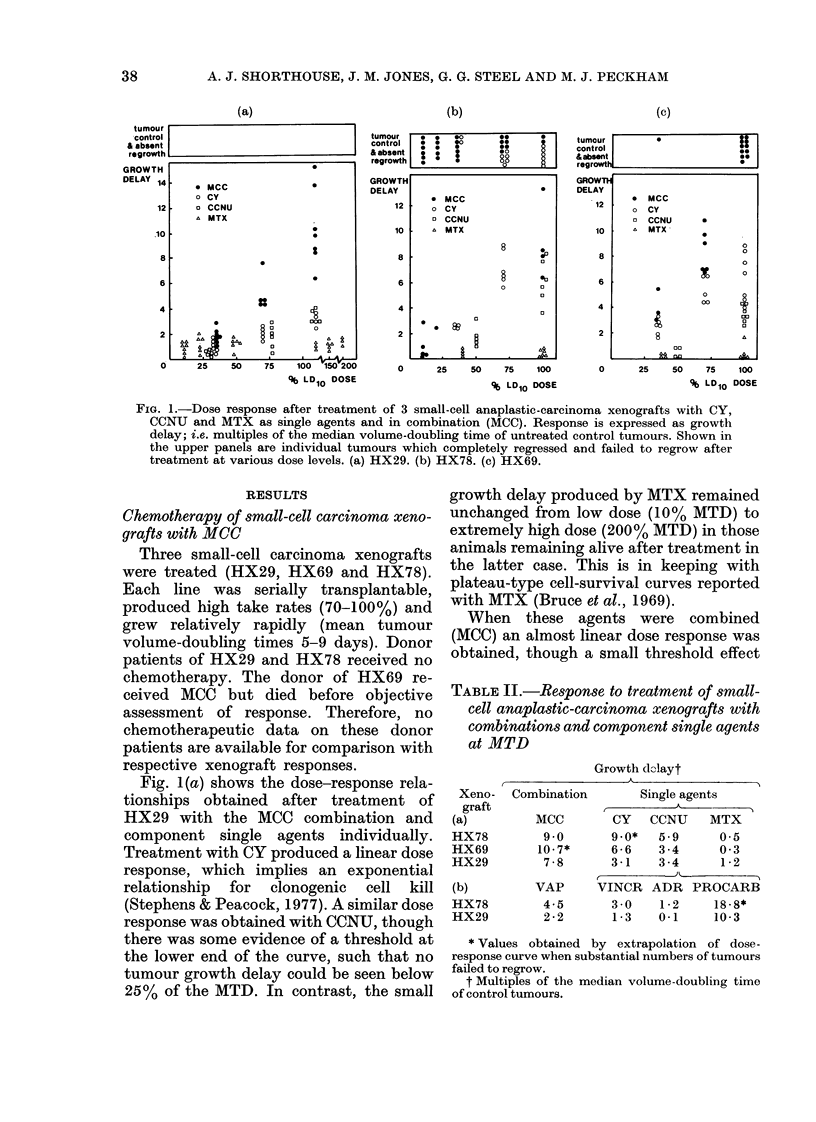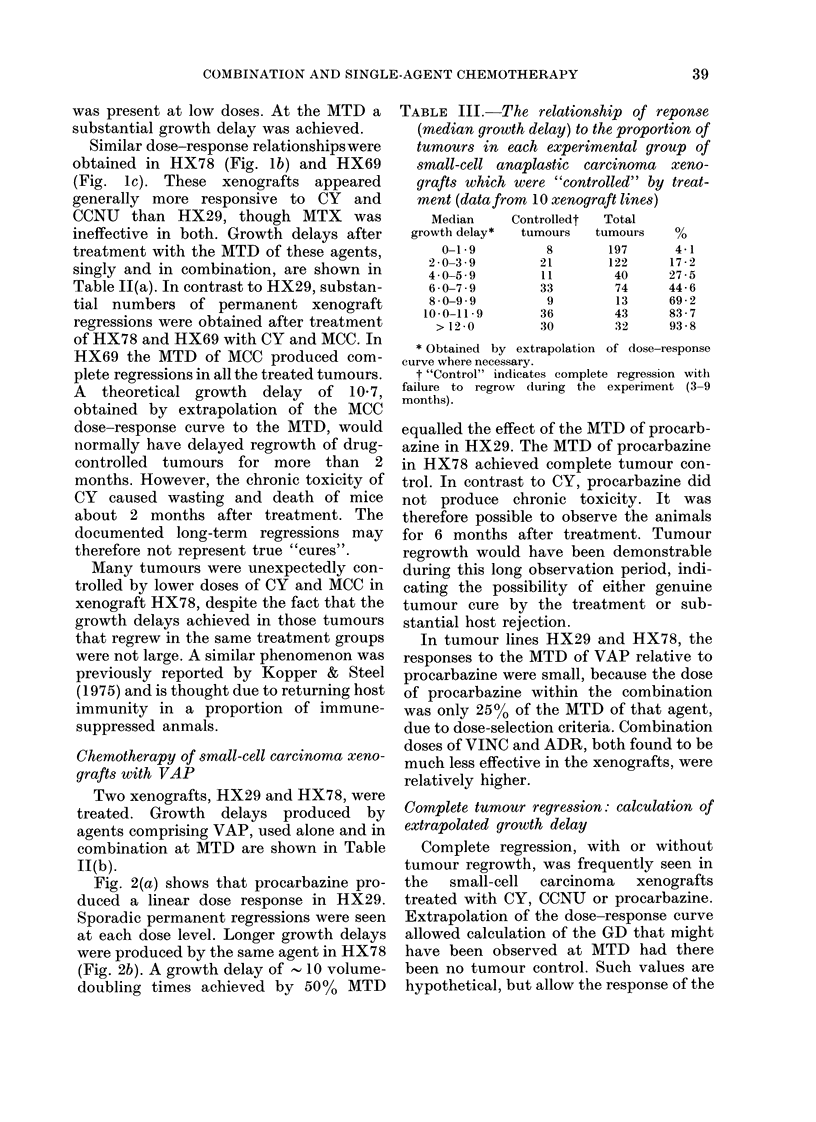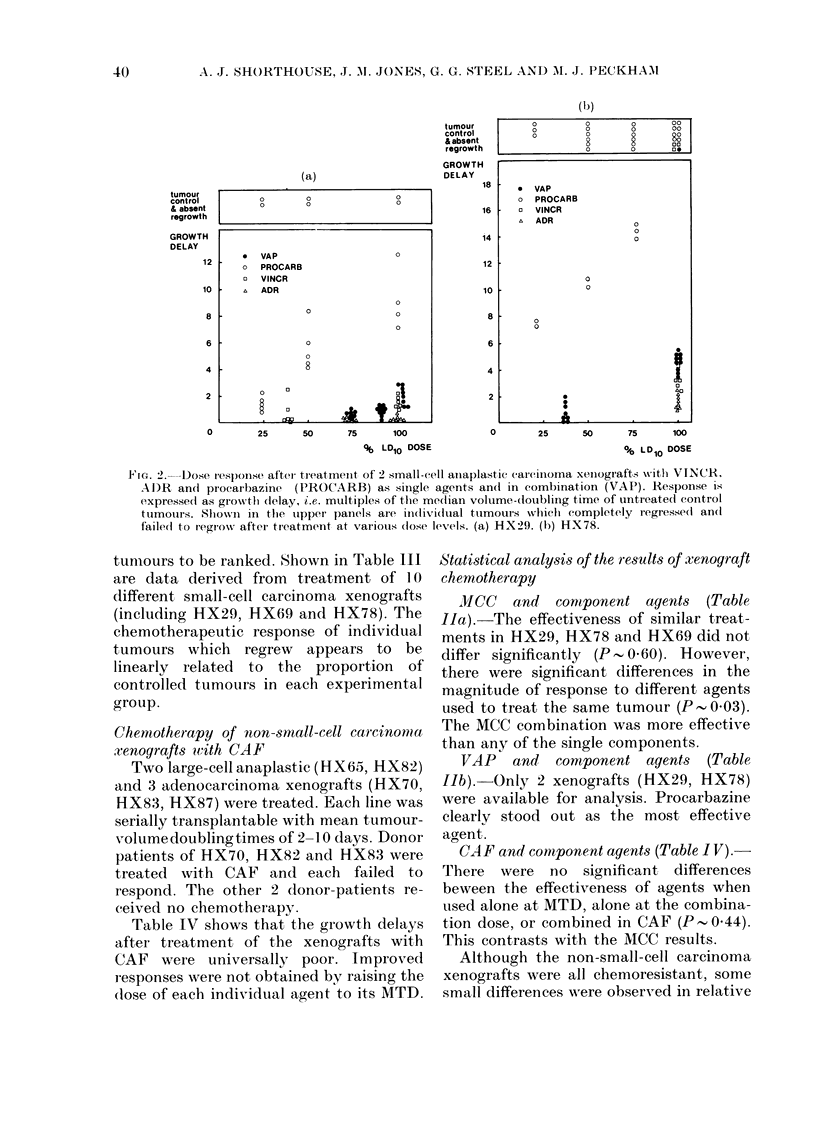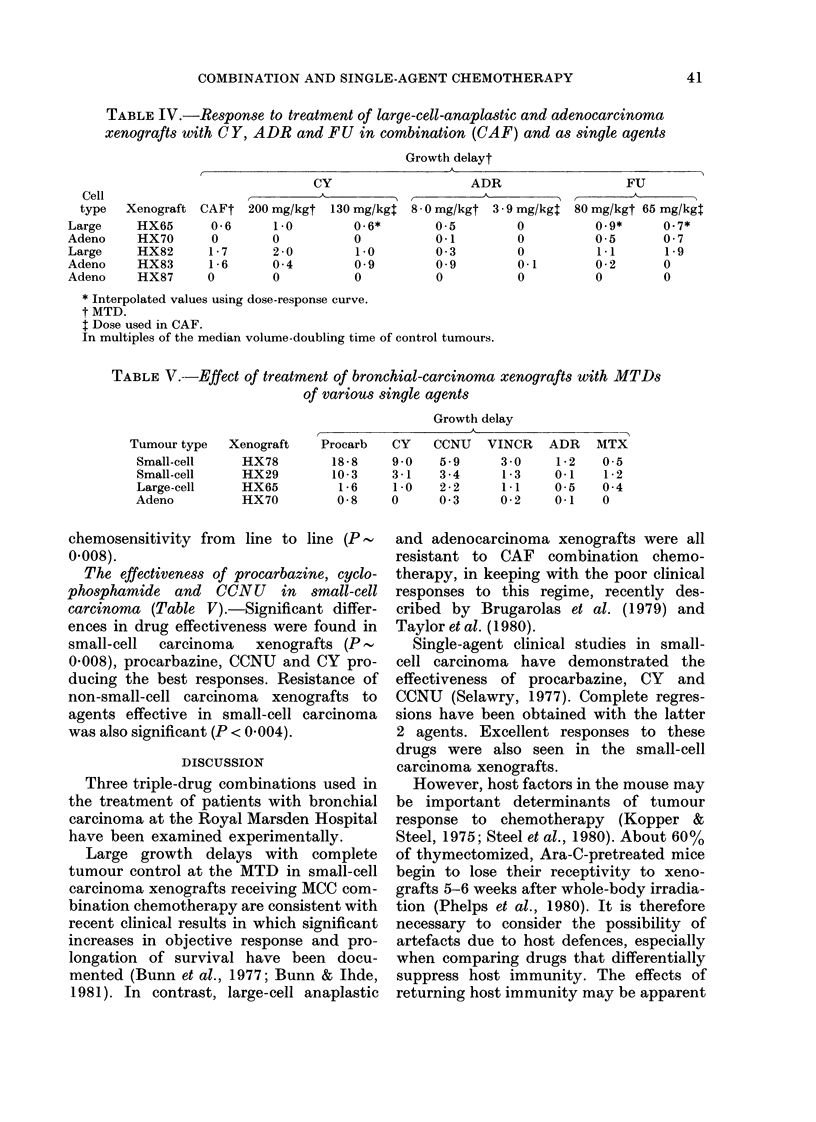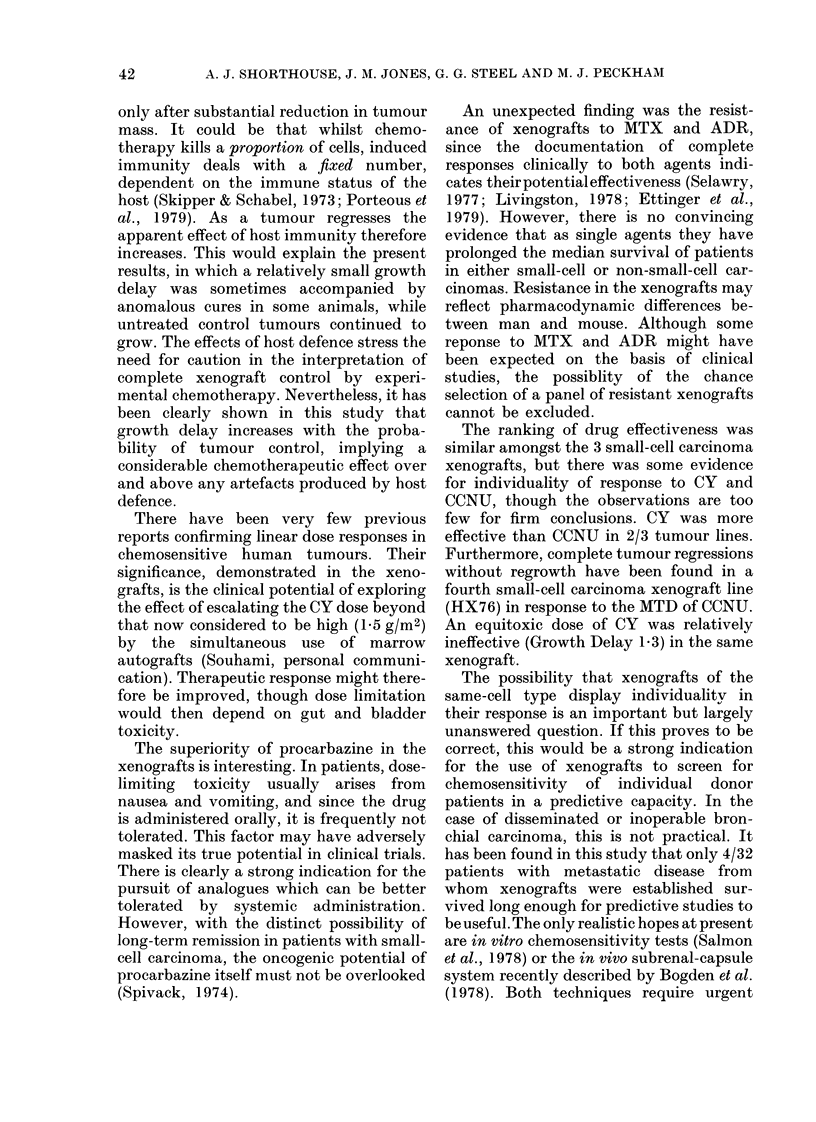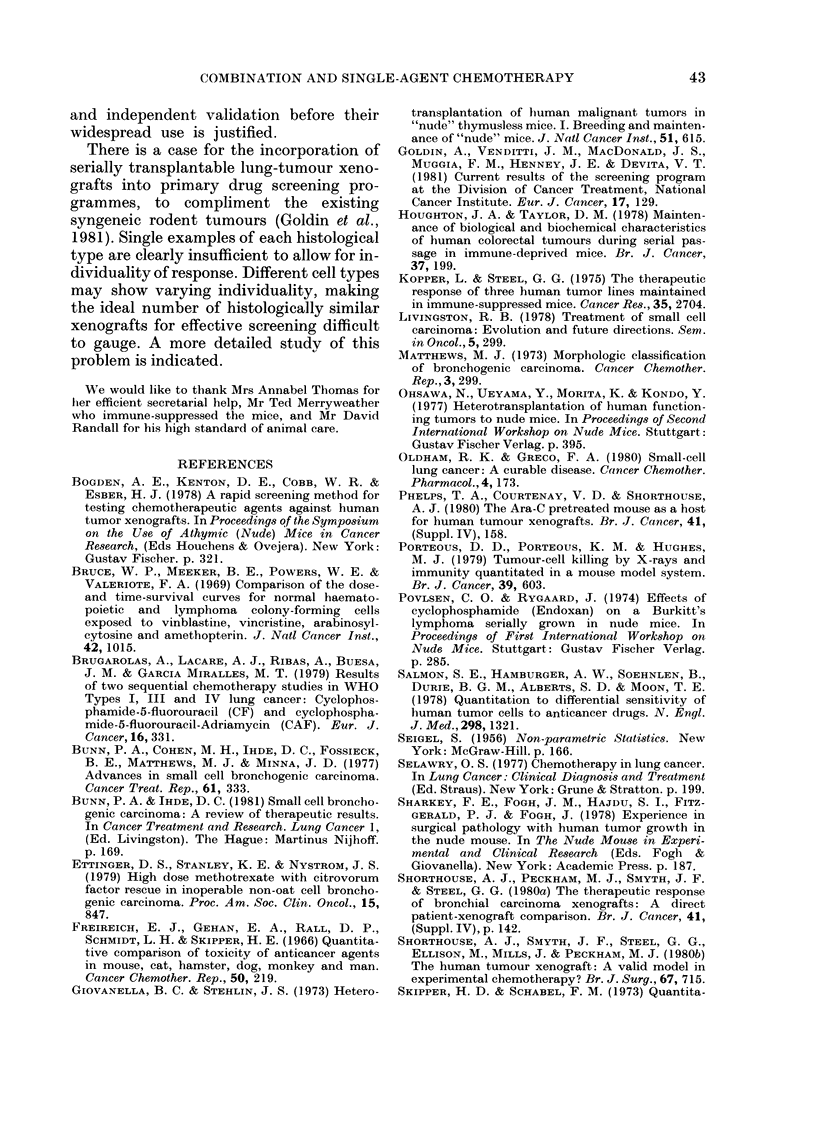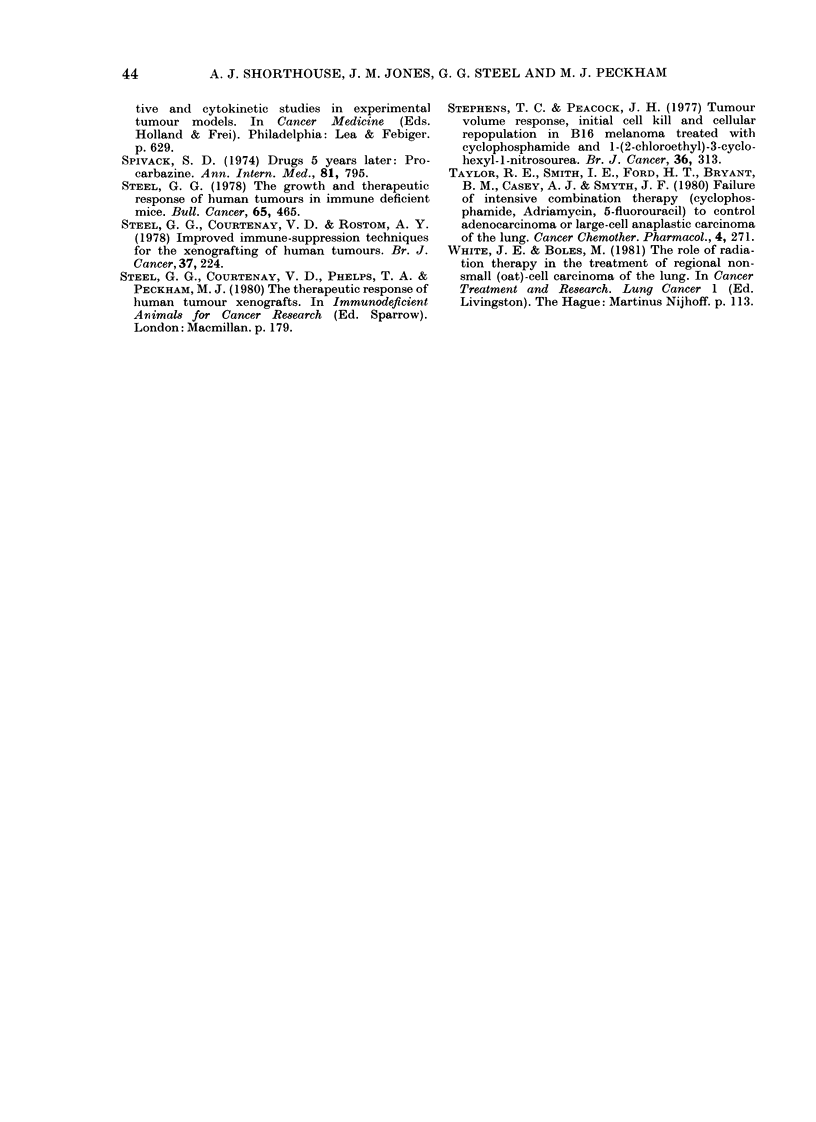# Experimental combination and single-agent chemotherapy in human lung-tumour xenografts.

**DOI:** 10.1038/bjc.1982.162

**Published:** 1982-07

**Authors:** A. J. Shorthouse, J. M. Jones, G. G. Steel, M. J. Peckham

## Abstract

A series of human bronchial-carcinoma xenografts (3 small-cell anaplastic, 2 large-cell anaplastic and 3 adenocarcinomas) established in immune-suppressed mice were treated with combination chemotherapy based on clinical regimes. Xenograft response was assessed by the in situ endpoint of growth delay in s.c. tumours. Dose-response relationships of 3 triple-drug combinations and their component agents were explored, allowing the relative contributions of single agents in each combination to be assessed. The results demonstrate that the effects produced in the xenografts were generally consistent with clinical experience. Procarbazine, cyclophosphamide and CCNU stood out as the most effective drugs in small cell carcinoma, but were ineffective in the other histological types. These was some evidence for individuality of therapeutic response among the grafts, supporting the case for incorporating panels of histologically similar xenografts into primary drug-screening programmes to complement existing syngeneic rodent tumour systems.


*Br. J. Cancer* (1982) **46**, 35

### EXPERIMENTAL COMBINATION AND SINGLE-AGENT CHEMOTHERAPY IN HUMAN LUNG-TUMOUR XENOGRAFTS

A. J. SHORTHOUSE*, J. M. JONES†, G. G. STEEL‡ AND M. J. PECKHAM*

*From the *Royal Marsden Hospital, Downs Road; the †Department of Epidemiology, and the ‡Radiotherapy Research Unit, Institute of Cancer Research, Clifton Avenue, Sutton, Surrey SM2 5PX*

Received 4 November 1981  Accepted 15 March 1982

**Summary.—A series of human bronchial-carcinoma xenografts (3 small-cell anaplastic, 2 large-cell anaplastic and 3 adenocarcinomas) established in immune-suppressed mice were treated with combination chemotherapy based on clinical regimes. Xenograft response was assessed by the *in situ* endpoint of growth delay in s.c. tumours. Dose–response relationships of 3 triple-drug combinations and their component agents were explored, allowing the relative contributions of single agents in each combination to be assessed. The results demonstrate that the effects produced in the xenografts were generally consistent with clinical experience. Procarbazine, cyclophosphamide and CCNU stood out as the most effective drugs in small cell carcinoma, but were ineffective in the other histological types.**

**There was some evidence for individuality of therapeutic response among the grafts, supporting the case for incorporating panels of histologically similar xenografts into primary drug-screening programmes to complement existing syngeneic rodent tumour systems.**

IMPROVEMENTS IN RESPONSE and prolongation of survival have been achieved with aggressive combination chemotherapy in small-cell anaplastic carcinoma of the lung. Some long-term remissions are now being obtained, but there still remains the problem of early relapse in many patients, with the appearance of disease that is refractory to further chemotherapy (Bunn *et al.*, 1977; Livingston, 1978; Oldham & Greco, 1980).

In contrast, there has been little progress in the treatment of advanced squamous, large-cell anaplastic and adenocarcinoma. Both single-agent and combination chemotherapy are associated with low response rates, with little evidence for prolonged survival (Selawry, 1977; White & Boles, 1981).

There is an urgent requirement, therefore, for more satisfactory laboratory tests to improve clinical results. The human tumour xenograft is an exciting development in experimental therapeutics, and may provide a more rational approach to the use of new and existing drugs in combination or as single agents in the treatment of bronchial carcinoma.

Morphology and functional activity of human tumours *in situ* appear to be largely retained when established as xenografts in nude or immune-suppressed rodents (Ohsawa *et al.*, 1977; Sharkey *et al.*, 1978; Steel, 1978; Houghton & Taylor, 1978).

Although xenografts have been found to respond to agents which are generally effective clinically (Povlsen & Rygaard, 1974; Kopper & Steel, 1975; Steel, 1978) we have recently established a more precise relationship between individual xenograft and donor–patient responses upon which the chemotherapeutic validity of xenografts ultimately depends. A total of 21

Correspondence to A. J. Shorthouse, 1 Coleshill Road, Teddington, Middlesex.

separate chemotherapeutic responses documented in 16 patients with bronchial carcinoma were similar to those found in their respective xenografts treated with the same single agents or combinations. Large differences were found between the chemosensitivity of most small-cell carcinomas and the chemoresistance of large-cell anaplastic, squamous and adenocarcinomas, demonstrating good parallelism between man and mouse among the histopathological categories of tumour (Shorthouse *et al.*, 1980*a*, *b*). Many of the patients in this study received combination chemotherapy, though an ideal comparison of xenograft and donor–patient responses would have involved the use of a single agent given to each patient and xenograft. However, the adoption of single-agent chemotherapy in patients with small-cell carcinoma specifically for a comparative xenograft study was considered unjustified, because of the clinical superiority of combination chemotherapy (Bunn *et al.*, 1977). Xenografts were therefore also treated with the same drug combinations received by individual donor patients.

An evaluation of the relative contribution of component drugs within these combinations is described in the present report. An attempt has been made to rank these agents in order of effectiveness against bronchial-carcinoma xenografts of different histologies.

### MATERIALS AND METHODS

Human bronchial carcinomas were established as s.c. xenografts in 8–10-week-old female CBA/lac mice immune-suppressed by neonatal thymectomy, cytosine arabinoside and whole-body irradiation (Steel *et al.*, 1978). Tumour-bearing mice were housed conventionally, 5/cage, and maintained on Spratt's No. 1 Rodent Breeding Diet and acidified water *ad libitum*. Specific-pathogen-free (SPF) conditions, necessary for optimum maintenance of nude mice (Giovanella & Stehlin, 1973) were not required.

Groups of tumours in early passage (2–11) received chemotherapy when their average volume reached 0·2–0·5 $cm^3$ (calculated by $\pi/6 \times$ mean diameter$^3$). The parameter of chemotherapeutic response was the *in situ* endpoint of growth delay, which was determined by dividing the difference between median volume-doubling times of control and treated tumours by the median doubling time of control tumours. This gave an estimate of growth delay due to treatment, in multiples of the doubling time of untreated tumours (Kopper & Steel, 1975).

Xenografts and original donor tumours were classified histologically according to the Working Party for Therapy of Lung Cancer (WP-L) classification (Matthews, 1973) and checked at each xenograft passage. Chromosome analysis was performed on xenografts to exclude the presence of syngeneic murine tumours.

Patients with small-cell carcinoma received cyclophosphamide (CY, 1 g/$m^2$) and CCNU (100 mg/$m^2$) combined with a 24h infusion of methotrexate (MTX, 200 mg/$m^2$) followed by folinic acid rescue (MCC). Two 6-week cycles of MCC were administered in the induction phase and then monthly courses of vincristine (VINCR, 1·4 mg/$m^2$), adriamycin (ADR, 40 mg/$m^2$) and procarbazine (100 mg/$m^2$) were given (VAP) for a total treatment period of about 9 months.

Patients with inoperable or advanced large-cell anaplastic or adenocarcinoma were treated with monthly cycles of CY (1 g/$m^2$), ADR (30 mg/$m^2$) and 5-fluorouracil (FU, 500 mg/$m^2$) in combination given on Days 1 and 8 (CAF).

In order to parallel the clinical situation experimentally, xenografts were given the same combination of agents used in the treatment of patients with similar tumour histology.

Ideally, chemotherapeutic agents should be given to mice in doses and schedules equivalent to those received by patients. However, since the question of comparative pharmacodynamics between species is intricate and data incomplete, there is at present no satisfactory basis for converting human to murine doses. The policy adopted was to use the maximum tolerated dose (MTD) in the mouse, determined by preliminary toxicity studies ($LD_{10}$ endpoint). Freireich *et al.* (1966) found that an MTD of drug correlated well from species to species on a mg/$m^2$ basis.

In order to assess dose–response relationships, combinations were given in increasing doses up to the MTD. The dose ratio of component agents within a combination used clinically was strictly maintained when treating the xenografts. Component drugs were therefore not always at equitoxic doses within the combinations, because their proportions were based on clinical doses rather than equal fractions of murine $LD_{10}$ doses.

To evaluate the relative contribution of component drugs within the MCC, VAP and CAF combinations, in terms of cytotoxicity, single-agent dose–response relationships were also studied.

Clinically, combination chemotherapy was given in repeated cycles. The xenografts, however, were restricted to a single cycle of treatment, which allowed tumour growth delay to be quantified and ranked amongst xenograft lines without interference from subsequent cycles of treatment. Since the doubling time, and the observable duration of tumour growth in the mouse is shorter than in man, it would have been difficult to adapt a prolonged repeated course of treatments to the mouse, and in the case of rapidly enlarging chemoresistant tumours, euthanasia of animals would have been necessary before completion of treatments.

Schedules of drug administration within a cycle were similar to those in patients. However, some compromises were necessary. For example, patients received MTX infused i.v. over 24 h, followed by folinic acid rescue, whereas mice were given 3 divided doses of MTX i.p. over 24 h. No folinic acid rescue was used in mice, because toxicity studies demonstrated that the MTD of the MCC combination contained only 40% of the MTD of MTX, so that folinic acid rescue would not have improved the survival of the animals.

Dosage and schedules for chemotherapeutic agents given to mice are shown in Table I. All agents were given i.p. in a volume of 0·01 ml/g of mouse body weight. MTX as a single agent or in combination was given in 3 divided doses over 24 h, in an attempt to mimic 24h infusion in patients. VINCR was given as a single injection on Days 1 and 5. Procarbazine was given in 5 equal daily fractions as single doses. All drugs were prepared in aqueous solution with the exception of CCNU, which was made up in 1 part 10% dimethyl sulphoxide (DMSO) in 9 parts 5 % Tween 80.

All agents were freshly prepared immediately before administration, with the exception of MTX which was stored at the appropriate concentration at −20°C.

*Statistical analysis.*—Chemotherapeutic responses of xenografts were expressed in terms of median growth delay and analysed by Friedman's 2-way analysis of variance by ranks (Seigel, 1956).

TABLE I.—*Chemotherapeutic agents in xenografts (all i.p.)*

| Agent | Manufacturer | Dose (mg/kg) Single* | Dose (mg/kg) Combined† | Schedule |
|---|---|---|---|---|
| Methotrexate (MTX) | Lederle | 100 | 40 (MCC) | 3 divided doses in 24 h |
| Cyclophosphamide (CY) | Koch–Light | 200 | 200 (MCC) 130 (CAF) | single dose |
| CCNU | Lundbeck | 40 | 20 (MCC) | single dose |
| 5-Fluorouracil (FU) | Roche | 80 | 65 (CAF) | single dose |
| Vincristine (VINCR) | Eli Lilly | 1·6 | 0·27 (VAP) | 0·8 mg/kg Days 1+5<br>0·135 mg/kg Days 1+5 |
| Adriamycin (ADR) | Mont Edison | 8·0 | 3·9 (CAF) 7·7 (VAP) | single dose |
| Procarbazine | Roche | 1300 | 270 (VAP) | 260 mg/kg o.d. x 5<br>54 mg/kg o.d. x 5 |

MCC = MTX/CY/CCNU in combination.
CAF = CY/ADR/FU in combination.
VAP = VINCR/ADR/procarbazine in combination.
* Single agent at a maximum tolerated dose (MTD).
† Dose of single agent in MTD of a combination (in parentheses).

FIG. 1.—Dose response after treatment of 3 small-cell anaplastic-carcinoma xenografts with CY, CCNU and MTX as single agents and in combination (MCC). Response is expressed as growth delay; *i.e.* multiples of the median volume-doubling time of untreated control tumours. Shown in the upper panels are individual tumours which completely regressed and failed to regrow after treatment at various dose levels. (a) HX29. (b) HX78. (c) HX69.

### RESULTS

### *Chemotherapy of small-cell carcinoma xenografts with MCC*

Three small-cell carcinoma xenografts were treated (HX29, HX69 and HX78). Each line was serially transplantable, produced high take rates (70–100%) and grew relatively rapidly (mean tumour volume-doubling times 5–9 days). Donor patients of HX29 and HX78 received no chemotherapy. The donor of HX69 received MCC but died before objective assessment of response. Therefore, no chemotherapeutic data on these donor patients are available for comparison with respective xenograft responses.

Fig. 1(*a*) shows the dose–response relationships obtained after treatment of HX29 with the MCC combination and component single agents individually. Treatment with CY produced a linear dose response, which implies an exponential relationship for clonogenic cell kill (Stephens & Peacock, 1977). A similar dose response was obtained with CCNU, though there was some evidence of a threshold at the lower end of the curve, such that no tumour growth delay could be seen below 25% of the MTD. In contrast, the small growth delay produced by MTX remained unchanged from low dose (10% MTD) to extremely high dose (200% MTD) in those animals remaining alive after treatment in the latter case. This is in keeping with plateau-type cell-survival curves reported with MTX (Bruce *et al.*, 1969).

When these agents were combined (MCC) an almost linear dose response was obtained, though a small threshold effect

TABLE II.—*Response to treatment of small-cell anaplastic-carcinoma xenografts with combinations and component single agents at MTD*

| | Growth delay† | | | |
|---|---|---|---|---|
| Xeno-graft | Combination | Single agents | | |
| (a) | MCC | CY | CCNU | MTX |
| HX78 | 9·0 | 9·0* | 5·9 | 0·5 |
| HX69 | 10·7* | 6·6 | 3·4 | 0·3 |
| HX29 | 7·8 | 3·1 | 3·4 | 1·2 |
| (b) | VAP | VINCR | ADR | PROCARB |
| HX78 | 4·5 | 3·0 | 1·2 | 18·8* |
| HX29 | 2·2 | 1·3 | 0·1 | 10·3 |

* Values obtained by extrapolation of dose-response curve when substantial numbers of tumours failed to regrow.

† Multiples of the median volume-doubling time of control tumours.

was present at low doses. At the MTD a substantial growth delay was achieved.

Similar dose–response relationships were obtained in HX78 (Fig. 1*b*) and HX69 (Fig. 1*c*). These xenografts appeared generally more responsive to CY and CCNU than HX29, though MTX was ineffective in both. Growth delays after treatment with the MTD of these agents, singly and in combination, are shown in Table II(a). In contrast to HX29, substantial numbers of permanent xenograft regressions were obtained after treatment of HX78 and HX69 with CY and MCC. In HX69 the MTD of MCC produced complete regressions in all the treated tumours. A theoretical growth delay of 10·7, obtained by extrapolation of the MCC dose–response curve to the MTD, would normally have delayed regrowth of drug-controlled tumours for more than 2 months. However, the chronic toxicity of CY caused wasting and death of mice about 2 months after treatment. The documented long-term regressions may therefore not represent true "cures".

Many tumours were unexpectedly controlled by lower doses of CY and MCC in xenograft HX78, despite the fact that the growth delays achieved in those tumours that regrew in the same treatment groups were not large. A similar phenomenon was previously reported by Kopper & Steel (1975) and is thought due to returning host immunity in a proportion of immune-suppressed anmals.

*Chemotherapy of small-cell carcinoma xenografts with VAP*

Two xenografts, HX29 and HX78, were treated. Growth delays produced by agents comprising VAP, used alone and in combination at MTD are shown in Table II(b).

Fig. 2(*a*) shows that procarbazine produced a linear dose response in HX29. Sporadic permanent regressions were seen at each dose level. Longer growth delays were produced by the same agent in HX78 (Fig. 2*b*). A growth delay of ~10 volume-doubling times achieved by 50% MTD equalled the effect of the MTD of procarbazine in HX29. The MTD of procarbazine in HX78 achieved complete tumour control. In contrast to CY, procarbazine did not produce chronic toxicity. It was therefore possible to observe the animals for 6 months after treatment. Tumour regrowth would have been demonstrable during this long observation period, indicating the possibility of either genuine tumour cure by the treatment or substantial host rejection.

TABLE III.—*The relationship of reponse (median growth delay) to the proportion of tumours in each experimental group of small-cell anaplastic carcinoma xenografts which were "controlled" by treatment (data from 10 xenograft lines)*

| Median growth delay* | Controlled† tumours | Total tumours | % |
|---|---|---|---|
| 0–1·9 | 8 | 197 | 4·1 |
| 2·0–3·9 | 21 | 122 | 17·2 |
| 4·0–5·9 | 11 | 40 | 27·5 |
| 6·0–7·9 | 33 | 74 | 44·6 |
| 8·0–9·9 | 9 | 13 | 69·2 |
| 10·0–11·9 | 36 | 43 | 83·7 |
| >12·0 | 30 | 32 | 93·8 |

* Obtained by extrapolation of dose–response curve where necessary.

† "Control" indicates complete regression with failure to regrow during the experiment (3–9 months).

In tumour lines HX29 and HX78, the responses to the MTD of VAP relative to procarbazine were small, because the dose of procarbazine within the combination was only 25% of the MTD of that agent, due to dose-selection criteria. Combination doses of VINC and ADR, both found to be much less effective in the xenografts, were relatively higher.

*Complete tumour regression: calculation of extrapolated growth delay*

Complete regression, with or without tumour regrowth, was frequently seen in the small-cell carcinoma xenografts treated with CY, CCNU or procarbazine. Extrapolation of the dose–response curve allowed calculation of the GD that might have been observed at MTD had there been no tumour control. Such values are hypothetical, but allow the response of the

FIG. 2.—Dose response after treatment of 2 small-cell anaplastic carcinoma xenografts with VINCR, ADR and procarbazine (PROCARB) as single agents and in combination (VAP). Response is expressed as growth delay, *i.e.* multiples of the median volume-doubling time of untreated control tumours. Shown in the upper panels are individual tumours which completely regressed and failed to regrow after treatment at various dose levels. (a) HX29. (b) HX78.

tumours to be ranked. Shown in Table III are data derived from treatment of 10 different small-cell carcinoma xenografts (including HX29, HX69 and HX78). The chemotherapeutic response of individual tumours which regrew appears to be linearly related to the proportion of controlled tumours in each experimental group.

*Chemotherapy of non-small-cell carcinoma xenografts with CAF*

Two large-cell anaplastic (HX65, HX82) and 3 adenocarcinoma xenografts (HX70, HX83, HX87) were treated. Each line was serially transplantable with mean tumour-volume doubling times of 2–10 days. Donor patients of HX70, HX82 and HX83 were treated with CAF and each failed to respond. The other 2 donor-patients received no chemotherapy.

Table IV shows that the growth delays after treatment of the xenografts with CAF were universally poor. Improved responses were not obtained by raising the dose of each individual agent to its MTD.

*Statistical analysis of the results of xenograft chemotherapy*

*MCC and component agents* (*Table IIa*).—The effectiveness of similar treatments in HX29, HX78 and HX69 did not differ significantly ($P \sim 0{\cdot}60$). However, there were significant differences in the magnitude of response to different agents used to treat the same tumour ($P \sim 0{\cdot}03$). The MCC combination was more effective than any of the single components.

*VAP and component agents* (*Table IIb*).—Only 2 xenografts (HX29, HX78) were available for analysis. Procarbazine clearly stood out as the most effective agent.

*CAF and component agents* (*Table IV*).—There were no significant differences beween the effectiveness of agents when used alone at MTD, alone at the combination dose, or combined in CAF ($P \sim 0{\cdot}44$). This contrasts with the MCC results.

Although the non-small-cell carcinoma xenografts were all chemoresistant, some small differences were observed in relative

TABLE IV.—*Response to treatment of large-cell-anaplastic and adenocarcinoma xenografts with CY, ADR and FU in combination (CAF) and as single agents*

| | | | Growth delay† | | | | | |
|---|---|---|---|---|---|---|---|---|
| | | | CY | | ADR | | FU | |
| Cell type | Xenograft | CAF† | 200 mg/kg† | 130 mg/kg‡ | 8·0 mg/kg† | 3·9 mg/kg‡ | 80 mg/kg† | 65 mg/kg‡ |
| Large | HX65 | 0·6 | 1·0 | 0·6* | 0·5 | 0 | 0·9* | 0·7* |
| Adeno | HX70 | 0 | 0 | 0 | 0·1 | 0 | 0·5 | 0·7 |
| Large | HX82 | 1·7 | 2·0 | 1·0 | 0·3 | 0 | 1·1 | 1·9 |
| Adeno | HX83 | 1·6 | 0·4 | 0·9 | 0·9 | 0·1 | 0·2 | 0 |
| Adeno | HX87 | 0 | 0 | 0 | 0 | 0 | 0 | 0 |

* Interpolated values using dose-response curve.
† MTD.
‡ Dose used in CAF.
In multiples of the median volume-doubling time of control tumours.

TABLE V.—*Effect of treatment of bronchial-carcinoma xenografts with MTDs of various single agents*

| | | Growth delay | | | | | |
|---|---|---|---|---|---|---|---|
| Tumour type | Xenograft | Procarb | CY | CCNU | VINCR | ADR | MTX |
| Small-cell | HX78 | 18·8 | 9·0 | 5·9 | 3·0 | 1·2 | 0·5 |
| Small-cell | HX29 | 10·3 | 3·1 | 3·4 | 1·3 | 0·1 | 1·2 |
| Large-cell | HX65 | 1·6 | 1·0 | 2·2 | 1·1 | 0·5 | 0·4 |
| Adeno | HX70 | 0·8 | 0 | 0·3 | 0·2 | 0·1 | 0 |

chemosensitivity from line to line ($P \sim 0{\cdot}008$).

*The effectiveness of procarbazine, cyclophosphamide and CCNU in small-cell carcinoma (Table V).*—Significant differences in drug effectiveness were found in small-cell carcinoma xenografts ($P \sim 0{\cdot}008$), procarbazine, CCNU and CY producing the best responses. Resistance of non-small-cell carcinoma xenografts to agents effective in small-cell carcinoma was also significant ($P < 0{\cdot}004$).

### DISCUSSION

Three triple-drug combinations used in the treatment of patients with bronchial carcinoma at the Royal Marsden Hospital have been examined experimentally.

Large growth delays with complete tumour control at the MTD in small-cell carcinoma xenografts receiving MCC combination chemotherapy are consistent with recent clinical results in which significant increases in objective response and prolongation of survival have been documented (Bunn *et al.*, 1977; Bunn & Ihde, 1981). In contrast, large-cell anaplastic and adenocarcinoma xenografts were all resistant to CAF combination chemotherapy, in keeping with the poor clinical responses to this regime, recently described by Brugarolas *et al.* (1979) and Taylor *et al.* (1980).

Single-agent clinical studies in small-cell carcinoma have demonstrated the effectiveness of procarbazine, CY and CCNU (Selawry, 1977). Complete regressions have been obtained with the latter 2 agents. Excellent responses to these drugs were also seen in the small-cell carcinoma xenografts.

However, host factors in the mouse may be important determinants of tumour response to chemotherapy (Kopper & Steel, 1975; Steel *et al.*, 1980). About 60% of thymectomized, Ara-C-pretreated mice begin to lose their receptivity to xenografts 5–6 weeks after whole-body irradiation (Phelps *et al.*, 1980). It is therefore necessary to consider the possibility of artefacts due to host defences, especially when comparing drugs that differentially suppress host immunity. The effects of returning host immunity may be apparent only after substantial reduction in tumour mass. It could be that whilst chemotherapy kills a *proportion* of cells, induced immunity deals with a *fixed* number, dependent on the immune status of the host (Skipper & Schabel, 1973; Porteous *et al.*, 1979). As a tumour regresses the apparent effect of host immunity therefore increases. This would explain the present results, in which a relatively small growth delay was sometimes accompanied by anomalous cures in some animals, while untreated control tumours continued to grow. The effects of host defence stress the need for caution in the interpretation of complete xenograft control by experimental chemotherapy. Nevertheless, it has been clearly shown in this study that growth delay increases with the probability of tumour control, implying a considerable chemotherapeutic effect over and above any artefacts produced by host defence.

There have been very few previous reports confirming linear dose responses in chemosensitive human tumours. Their significance, demonstrated in the xenografts, is the clinical potential of exploring the effect of escalating the CY dose beyond that now considered to be high (1·5 g/m$^2$) by the simultaneous use of marrow autografts (Souhami, personal communication). Therapeutic response might therefore be improved, though dose limitation would then depend on gut and bladder toxicity.

The superiority of procarbazine in the xenografts is interesting. In patients, dose-limiting toxicity usually arises from nausea and vomiting, and since the drug is administered orally, it is frequently not tolerated. This factor may have adversely masked its true potential in clinical trials. There is clearly a strong indication for the pursuit of analogues which can be better tolerated by systemic administration. However, with the distinct possibility of long-term remission in patients with small-cell carcinoma, the oncogenic potential of procarbazine itself must not be overlooked (Spivack, 1974).

An unexpected finding was the resistance of xenografts to MTX and ADR, since the documentation of complete responses clinically to both agents indicates their potential effectiveness (Selawry, 1977; Livingston, 1978; Ettinger *et al.*, 1979). However, there is no convincing evidence that as single agents they have prolonged the median survival of patients in either small-cell or non-small-cell carcinomas. Resistance in the xenografts may reflect pharmacodynamic differences between man and mouse. Although some reponse to MTX and ADR might have been expected on the basis of clinical studies, the possiblity of the chance selection of a panel of resistant xenografts cannot be excluded.

The ranking of drug effectiveness was similar amongst the 3 small-cell carcinoma xenografts, but there was some evidence for individuality of response to CY and CCNU, though the observations are too few for firm conclusions. CY was more effective than CCNU in 2/3 tumour lines. Furthermore, complete tumour regressions without regrowth have been found in a fourth small-cell carcinoma xenograft line (HX76) in response to the MTD of CCNU. An equitoxic dose of CY was relatively ineffective (Growth Delay 1·3) in the same xenograft.

The possibility that xenografts of the same-cell type display individuality in their response is an important but largely unanswered question. If this proves to be correct, this would be a strong indication for the use of xenografts to screen for chemosensitivity of individual donor patients in a predictive capacity. In the case of disseminated or inoperable bronchial carcinoma, this is not practical. It has been found in this study that only 4/32 patients with metastatic disease from whom xenografts were established survived long enough for predictive studies to be useful. The only realistic hopes at present are *in vitro* chemosensitivity tests (Salmon *et al.*, 1978) or the *in vivo* subrenal-capsule system recently described by Bogden *et al.* (1978). Both techniques require urgent and independent validation before their widespread use is justified.

There is a case for the incorporation of serially transplantable lung-tumour xenografts into primary drug screening programmes, to compliment the existing syngeneic rodent tumours (Goldin *et al.*, 1981). Single examples of each histological type are clearly insufficient to allow for individuality of response. Different cell types may show varying individuality, making the ideal number of histologically similar xenografts for effective screening difficult to gauge. A more detailed study of this problem is indicated.

We would like to thank Mrs Annabel Thomas for her efficient secretarial help, Mr Ted Merryweather who immune-suppressed the mice, and Mr David Randall for his high standard of animal care.

REFERENCES

Bogden, A. E., Kenton, D. E., Cobb, W. R. & Esber, H. J. (1978) A rapid screening method for testing chemotherapeutic agents against human tumor xenografts. In *Proceedings of the Symposium on the Use of Athymic (Nude) Mice in Cancer Research*, (Eds Houchens & Ovejera). New York: Gustav Fischer. p. 321.

Bruce, W. P., Meeker, B. E., Powers, W. E. & Valeriote, F. A. (1969) Comparison of the dose- and time-survival curves for normal haematopoietic and lymphoma colony-forming cells exposed to vinblastine, vincristine, arabinosylcytosine and amethopterin. *J. Natl Cancer Inst.*, **42**, 1015.

Brugarolas, A., Lacare, A. J., Ribas, A., Buesa, J. M. & Garcia Miralles, M. T. (1979) Results of two sequential chemotherapy studies in WHO Types I, III and IV lung cancer: Cyclophosphamide-5-fluorouracil (CF) and cyclophosphamide-5-fluorouracil-Adriamycin (CAF). *Eur. J. Cancer*, **16**, 331.

Bunn, P. A., Cohen, M. H., Ihde, D. C., Fossieck, B. E., Matthews, M. J. & Minna, J. D. (1977) Advances in small cell bronchogenic carcinoma. *Cancer Treat. Rep.*, **61**, 333.

Bunn, P. A. & Ihde, D. C. (1981) Small cell bronchogenic carcinoma: A review of therapeutic results. In *Cancer Treatment and Research. Lung Cancer* 1, (Ed. Livingston). The Hague: Martinus Nijhoff. p. 169.

Ettinger, D. S., Stanley, K. E. & Nystrom, J. S. (1979) High dose methotrexate with citrovorum factor rescue in inoperable non-oat cell bronchogenic carcinoma. *Proc. Am. Soc. Clin. Oncol.*, **15**, 847.

Freireich, E. J., Gehan, E. A., Rall, D. P., Schmidt, L. H. & Skipper, H. E. (1966) Quantitative comparison of toxicity of anticancer agents in mouse, cat, hamster, dog, monkey and man. *Cancer Chemother. Rep.*, **50**, 219.

Giovanella, B. C. & Stehlin, J. S. (1973) Heterotransplantation of human malignant tumors in "nude" thymusless mice. I. Breeding and maintenance of "nude" mice. *J. Natl Cancer Inst.*, **51**, 615.

Goldin, A., Venditti, J. M., MacDonald, J. S., Muggia, F. M., Henney, J. E. & Devita, V. T. (1981) Current results of the screening program at the Division of Cancer Treatment, National Cancer Institute. *Eur. J. Cancer*, **17**, 129.

Houghton, J. A. & Taylor, D. M. (1978) Maintenance of biological and biochemical characteristics of human colorectal tumours during serial passage in immune-deprived mice. *Br. J. Cancer*, **37**, 199.

Kopper, L. & Steel, G. G. (1975) The therapeutic response of three human tumor lines maintained in immune-suppressed mice. *Cancer Res.*, **35**, 2704.

Livingston, R. B. (1978) Treatment of small cell carcinoma: Evolution and future directions. *Sem. in Oncol.*, **5**, 299.

Matthews, M. J. (1973) Morphologic classification of bronchogenic carcinoma. *Cancer Chemother. Rep.*, **3**, 299.

Ohsawa, N., Ueyama, Y., Morita, K. & Kondo, Y. (1977) Heterotransplantation of human functioning tumors to nude mice. In *Proceedings of Second International Workshop on Nude Mice*. Stuttgart: Gustav Fischer Verlag. p. 395.

Oldham, R. K. & Greco, F. A. (1980) Small-cell lung cancer: A curable disease. *Cancer Chemother. Pharmacol.*, **4**, 173.

Phelps, T. A., Courtenay, V. D. & Shorthouse, A. J. (1980) The Ara-C pretreated mouse as a host for human tumour xenografts. *Br. J. Cancer*, **41**, (Suppl. IV), 158.

Porteous, D. D., Porteous, K. M. & Hughes, M. J. (1979) Tumour-cell killing by X-rays and immunity quantitated in a mouse model system. *Br. J. Cancer*, **39**, 603.

Povlsen, C. O. & Rygaard, J. (1974) Effects of cyclophosphamide (Endoxan) on a Burkitt's lymphoma serially grown in nude mice. In *Proceedings of First International Workshop on Nude Mice*. Stuttgart: Gustav Fischer Verlag. p. 285.

Salmon, S. E., Hamburger, A. W., Soehnlen, B., Durie, B. G. M., Alberts, S. D. & Moon, T. E. (1978) Quantitation to differential sensitivity of human tumor cells to anticancer drugs. *N. Engl. J. Med.*, **298**, 1321.

Seigel, S. (1956) *Non-parametric Statistics*. New York: McGraw-Hill. p. 166.

Selawry, O. S. (1977) Chemotherapy in lung cancer. In *Lung Cancer: Clinical Diagnosis and Treatment* (Ed. Straus). New York: Grune & Stratton. p. 199.

Sharkey, F. E., Fogh, J. M., Hajdu, S. I., Fitzgerald, P. J. & Fogh, J. (1978) Experience in surgical pathology with human tumor growth in the nude mouse. In *The Nude Mouse in Experimental and Clinical Research* (Eds. Fogh & Giovanella). New York: Academic Press. p. 187.

Shorthouse, A. J., Peckham, M. J., Smyth, J. F. & Steel, G. G. (1980*a*) The therapeutic response of bronchial carcinoma xenografts: A direct patient-xenograft comparison. *Br. J. Cancer*, **41**, (Suppl. IV), p. 142.

Shorthouse, A. J., Smyth, J. F., Steel, G. G., Ellison, M., Mills, J. & Peckham, M. J. (1980*b*) The human tumour xenograft: A valid model in experimental chemotherapy? *Br. J. Surg.*, **67**, 715.

Skipper, H. D. & Schabel, F. M. (1973) Quantitative and cytokinetic studies in experimental tumour models. In *Cancer Medicine* (Eds. Holland & Frei). Philadelphia: Lea & Febiger. p. 629.

SPIVACK, S. D. (1974) Drugs 5 years later: Procarbazine. *Ann. Intern. Med.*, **81,** 795.

STEEL, G. G. (1978) The growth and therapeutic response of human tumours in immune deficient mice. *Bull. Cancer*, **65,** 465.

STEEL, G. G., COURTENAY, V. D. & ROSTOM, A. Y. (1978) Improved immune-suppression techniques for the xenografting of human tumours. *Br. J. Cancer*, **37,** 224.

STEEL, G. G., COURTENAY, V. D., PHELPS, T. A. & PECKHAM, M. J. (1980) The therapeutic response of human tumour xenografts. In *Immunodeficient Animals for Cancer Research* (Ed. Sparrow). London: Macmillan. p. 179.

STEPHENS, T. C. & PEACOCK, J. H. (1977) Tumour volume response, initial cell kill and cellular repopulation in B16 melanoma treated with cyclophosphamide and 1-(2-chloroethyl)-3-cyclohexyl-1-nitrosourea. *Br. J. Cancer*, **36,** 313.

TAYLOR, R. E., SMITH, I. E., FORD, H. T., BRYANT, B. M., CASEY, A. J. & SMYTH, J. F. (1980) Failure of intensive combination therapy (cyclophosphamide, Adriamycin, 5-fluorouracil) to control adenocarcinoma or large-cell anaplastic carcinoma of the lung. *Cancer Chemother. Pharmacol.*, **4,** 271.

WHITE, J. E. & BOLES, M. (1981) The role of radiation therapy in the treatment of regional non-small (oat)-cell carcinoma of the lung. In *Cancer Treatment and Research. Lung Cancer* 1 (Ed. Livingston). The Hague: Martinus Nijhoff. p. 113.